# A Comparative Analysis of Clinical Features of Type 2 Diabetes Mellitus With Respect to Dyslipidemia: A Cross-Sectional Study

**DOI:** 10.7759/cureus.101239

**Published:** 2026-01-10

**Authors:** Iqra Shareef, Ushaiqa Akbar, Marwah Usmani, Shireen Fatima, FNU Inayatullah, Faisal Iqbal, Adnan Anwar, Muhammad Irfan, Atif A Hashmi

**Affiliations:** 1 Medicine, Karachi Institute of Medical Sciences, Karachi, PAK; 2 Medicine, Hamdard College of Medicine and Dentistry, Karachi, PAK; 3 Family Medicine, Islam Medical College, Sialkot, PAK; 4 Physiology, Hamdard College of Medicine and Dentistry, Karachi, PAK; 5 Internal Medicine, Sindh Government Hospital, Karachi, PAK; 6 Statistics, Liaquat National Hospital and Medical College, Karachi, PAK; 7 Pathology, Indus Hospital and Health Network, Karachi, PAK

**Keywords:** clinical symptoms, diabetes, diabetes mellitus, diabetes treatment, dyslipidemia

## Abstract

Background: Diabetes mellitus type 2 is frequently associated with dyslipidemia, which accelerates cardiovascular risk and worsens metabolic control. Understanding how clinical features differ between patients with diabetes with and without dyslipidemia is essential for early identification of high-risk individuals.

Objective: To compare the clinical features of patients with type 2 diabetes who have dyslipidemia and those who do not, to identify any important differences in their health patterns.

Methods: This cross-sectional study was conducted over six months (March-August 2024) at multiple secondary and primary care centers in Karachi, Pakistan, with ethical approval from Sindh Government Hospital, Malir (Approval No. 1477). A total of 340 patients aged 30-75 years were enrolled, divided equally into dyslipidemic (Group A) and non-dyslipidemic (Group B) groups. The chi-square test and an independent t-test were used to examine associations between categorical and continuous variables. Pearson’s chi-square statistic, degrees of freedom, and p-values were obtained. Effect size was calculated using Cramer’s V to determine the strength of association.

Results: The dyslipidemia group showed distinct demographic patterns with male predominance (117 (68.8%), p < 0.001), higher smoking prevalence (45.3% vs 17.1%, p < 0.001), and lower physical activity levels (61.2% vs 73.5%, p = 0.015). These patients were significantly older (60.0 ± 15.85 vs 53.51 ± 14.40 years, p < 0.001) and had greater weight (72.05 ± 13.07 vs 66.90 ± 16.01 kg, p = 0.001). Clinical symptoms were more prevalent among dyslipidemic patients, including frequent urination (99 (58.2%) vs 46 (27.1%), p < 0.001), visual impairment (104 (61.2%) vs 72 (42.4%), p = 0.001), and blurred vision (78 (45.9%) vs 47 (27.6%), p < 0.001).

Conclusion: This study concluded that patients with type 2 diabetes mellitus and dyslipidemia had a higher prevalence of visual disturbances, urinary symptoms, fatigue, musculoskeletal pain, and other systemic complaints compared with non-dyslipidemic patients. Significant differences were observed in blurred vision, chest discomfort, increased thirst, fatigue, and mood changes.

## Introduction

Type 2 diabetes mellitus (T2DM) represents a significant global health challenge, affecting approximately 537 million adults worldwide in 2021, with projections suggesting an increase to 783 million by 2045 [1]. The escalating prevalence is particularly concerning in developing countries, where rapid urbanization and lifestyle changes contribute to increasing obesity rates and insulin resistance [2]. The complex interplay between T2DM and dyslipidemia has garnered substantial attention, as both conditions significantly contribute to cardiovascular complications and overall morbidity [3].

Dyslipidemia, characterized by abnormal lipid profiles, affects approximately 72-85% of patients with T2DM, substantially increasing their risk of cardiovascular events [4]. The characteristic pattern includes elevated triglycerides (TG), decreased high-density lipoprotein cholesterol (HDL-C), and increased small dense low-density lipoprotein (LDL-C) [5]. This atherogenic dyslipidemia profile significantly enhances the risk of macrovascular complications and accelerates atherosclerosis in patients with diabetes [6].

The coexistence of T2DM and dyslipidemia is associated with a distinct clinical profile, including various microvascular and macrovascular complications [7]. Current evidence suggests that patients with both conditions experience more severe symptoms and complications compared with those with T2DM alone, including a higher incidence of retinopathy, nephropathy, and neuropathy [8]. These manifestations often include visual disturbances, peripheral neuropathy, and accelerated cardiovascular disease progression, with the presence of dyslipidemia in patients with T2DM associated with a 2-4-fold increased risk of cardiovascular mortality [9].

Despite advances in understanding these conditions, there remains a need for comprehensive analysis of the clinical features distinguishing patients with T2DM with and without dyslipidemia. Recent studies have highlighted the importance of early identification and management of dyslipidemia in patients with T2DM to prevent complications and improve outcomes [10].

Although numerous studies have explored the association between T2DM and dyslipidemia, variations in clinical presentation across different populations remain underreported. Lifestyle, diet, and genetic factors can influence how dyslipidemia develops and its severity in patients with type 2 diabetes. To better understand these differences in the Pakistani population, this cross-sectional study was conducted to compare the clinical features of patients with diabetes with and without dyslipidemia. The findings aim to provide useful insights for more personalized prevention and treatment strategies for this population.

## Materials and methods

Study design and duration

This cross-sectional study was conducted over a six-month period, from March 1, 2024, to August 31, 2024, using a non-probability sampling technique at multiple secondary care centers and primary care clinics in Karachi, Pakistan. The centers included the Urban Health Center, Malir, the Urban Health Center, North Karachi, and Maymar Medical Center, Karachi.

Ethical statement

Ethical approval was obtained from the Ethical Review Board of Sindh Government Hospital, Malir, Karachi, Pakistan (Approval No. 1477).

Sample size calculation

The sample size was calculated using the WHO-recommended formula for prevalence studies (prevalence = 72%):

$n = \frac{Z^{2} &times; p &times; q}{d^{2}}$

where Z = 1.96 (95% confidence level), p = 72% = 0.72, q = 1 − p = 0.28, and d = 5% = 0.05.

The minimum required sample size was 310 participants, assuming a prevalence of 72% [4], a 95% confidence level, and a 5% margin of error.

Study population

A total of 340 patients with T2DM aged 30 to 75 years were included and divided into two equal groups. Group A consisted of 170 patients with dyslipidemia, a condition characterized by abnormal levels of blood lipids, including elevated total cholesterol, low-density lipoprotein cholesterol (LDL-C), triglycerides, or reduced high-density lipoprotein cholesterol (HDL-C), as defined by the National Cholesterol Education Program Adult Treatment Panel III (NCEP-ATP III) guidelines [11]. Group B included 170 patients without dyslipidemia. However, patients with secondary causes of dyslipidemia such as hypothyroidism, nephrotic syndrome, or chronic liver disease, those receiving medications affecting lipid metabolism (e.g., statins, corticosteroids, beta-blockers, or oral contraceptives), as well as individuals with acute or chronic infections, severe systemic illnesses, type 1 diabetes mellitus, gestational diabetes, or other endocrine disorders were excluded from the study.

Study measures

Demographic data, including age, gender, socioeconomic status, overall health status, comorbid conditions, and diabetes-related symptoms, were collected. All participants had T2DM, a chronic metabolic disorder marked by high blood sugar resulting from insulin resistance and relative insulin deficiency, typically diagnosed by a glycated hemoglobin (HbA1c) level of ≥6.5% according to the American Diabetes Association (ADA) criteria [12]. To identify patients with T2DM, HbA1c levels from the past six months were used. Anthropometric measurements such as height and weight were used to calculate body mass index (BMI) to assess obesity.

Lipid parameters, including total cholesterol, LDL-C, HDL-C, and triglycerides, were measured according to the National Cholesterol Education Program Adult Treatment Panel III (NCEP-ATP III) guidelines [11], defined as total cholesterol ≥200 mg/dL, LDL-C ≥130 mg/dL, HDL-C <40 mg/dL in men or <50 mg/dL in women, or triglycerides ≥150 mg/dL. Psychological health was assessed using structured questions designed to evaluate signs of depression, anxiety, and tension. Psychological disturbances such as depression and anxiety have been shown to worsen metabolic control, increase the risk of complications, and reduce quality of life in patients with diabetes, making this assessment clinically relevant.

A structured questionnaire was also administered to collect information on current medical history and previous sleep disturbances, including insomnia, difficulty initiating or maintaining sleep, and abnormal sleep behaviors. Sleep quality is an important parameter because disrupted sleep patterns have been associated with poor glycemic control, increased insulin resistance, and a higher prevalence of cardiovascular complications in patients with T2DM. By systematically recording these disturbances, the study explored potential links between sleep patterns, metabolic health, and the presence of dyslipidemia.

Ocular symptoms indicative of dry eye disease were evaluated through patient-reported experiences such as eye soreness, gritty or foreign body sensation, irritation, inflammation, and transient blurred vision that improved with blinking or tear secretion. These subjective measures, although not as precise as clinical ophthalmic tests, are relevant because dry eye disease is commonly reported in patients with T2DM and may reflect microvascular complications, poor glycemic control, or systemic inflammation. Early identification of ocular discomfort can prompt further ophthalmologic evaluation and improve patient outcomes.

Additionally, physiological parameters, including heart rate and random blood glucose levels, were recorded. Heart rate provides insight into autonomic nervous system function, which can be altered in diabetic neuropathy, while random blood glucose levels reflect short-term glycemic status and may identify patients with poorly controlled diabetes. Together, these measurements complement clinical and laboratory assessments, allowing a more comprehensive characterization of participants’ overall health and helping to identify correlations between metabolic, psychological, and ocular parameters in patients with and without dyslipidemia.

Data analysis

Data were analyzed using IBM SPSS Statistics for Windows, Version 26 (Released 2018; IBM Corp., Armonk, New York). Categorical variables, including sociodemographic characteristics and clinical symptoms, were reported as frequencies and percentages, while continuous variables were expressed as means ± standard deviations. The chi-square test was used to examine associations between categorical variables. Pearson’s chi-square statistic, degrees of freedom, and p-values were obtained. Effect size was calculated using Cramer’s V to determine the strength of association. Additionally, the independent t-test and Mann-Whitney test were used to compare mean values of continuous variables between groups, and a p-value of <0.05 was considered statistically significant.

## Results

Demographic characteristics

Table 1 shows the demographic characteristics of patients with T2DM in relation to their history of dyslipidemia. Among patients with dyslipidemia, 117 (68.8%) were male and 53 (31.2%) were female, whereas among patients without dyslipidemia, 43 (25.3%) were male and 127 (74.7%) were female, showing a significant association (p < 0.001). Regarding socioeconomic status, 28 (16.5%) patients with dyslipidemia and 31 (18.2%) patients without dyslipidemia belonged to the low-income group; 86 (50.6%) with dyslipidemia and 101 (59.4%) without dyslipidemia were from the middle-income group; and 56 (32.9%) with dyslipidemia and 38 (22.4%) without dyslipidemia were from the high-income group, with no significant association observed (p = 0.091). A history of hypertension was present in 124 (72.9%) patients with dyslipidemia compared with 117 (68.8%) patients without dyslipidemia, while 46 (27.1%) and 53 (31.2%), respectively, had no history of hypertension (p = 0.403). Smoking history showed a strong association, with 77 (45.3%) patients with dyslipidemia reporting smoking compared with 29 (17.1%) patients without dyslipidemia; conversely, 93 (54.7%) and 141 (82.9%), respectively, reported no smoking history (p < 0.001). Physical activity was reported by 104 (61.2%) patients with dyslipidemia and 125 (73.5%) patients without dyslipidemia, whereas 66 (38.8%) and 45 (26.5%), respectively, reported no physical activity, showing a significant association (p = 0.015).

**Table 1:** Demographic details of patients with type 2 diabetes mellitus with respect to history of dyslipidemia.

Variables	Category	History of dyslipidemia		Pearson chi square	degrees of freedom (df)	Effect size (Cramer’s V)	P-value
		Yes, n (%)	No, n (%)				
Gender	Male	117 (68.8%)	43 (25.3%)	202.723	1	0.772	<0.001
	Female	53 (31.2%)	127 (74.7%)				
Socioeconomic status	Low	28 (16.5%)	31 (18.2%)	4.803	2	0.119	0.091
	Middle	86 (50.6%)	101 (59.4%)				
	High	56 (32.9%)	38 (22.4%)				
History of hypertension (≥140/90 mmHg)	Yes	124 (72.9%)	117 (68.8%)	0.698	1	0.045	0.403
	No	46 (27.1%)	53 (31.2%)				
History of smoking	Yes	77 (45.3%)	29 (17.1%)	31.582	1	0.305	<0.001
	No	93 (54.7%)	141 (82.9%)				
Physical activity	Yes	104 (61.2%)	125 (73.5%)	5.899	1	0.132	0.015
	No	66 (38.8%)	45 (26.5%)				

Clinical parameters

Table 2 presents the comparison of clinical parameters between patients with and without a history of dyslipidemia. The mean age of patients with dyslipidemia was 60.0 ± 15.85 years, which was significantly higher than 53.51 ± 14.40 years in patients without dyslipidemia (p < 0.001). The dyslipidemia group also had a higher mean weight of 72.05 ± 13.07 kg compared with 66.90 ± 16.01 kg in the non-dyslipidemia group (p = 0.001). Mean height was greater among patients with dyslipidemia (68.09 ± 10.88 inches) compared with those without dyslipidemia (64.67 ± 7.43 inches), showing a significant difference (p = 0.001). Respiratory rate was also higher in the dyslipidemia group at 20.27 ± 5.97 cycles/min compared with 18.30 ± 5.58 cycles/min in patients without dyslipidemia (p = 0.002). Similarly, mean heart rate was significantly higher among patients with dyslipidemia (87.68 ± 10.46 beats/min) than among patients without dyslipidemia (82.62 ± 12.02 beats/min) (p < 0.001). Random blood sugar (RBS) levels were 335.88 ± 97.64 mg/dL in the dyslipidemia group and 320.70 ± 111.99 mg/dL in the non-dyslipidemia group; however, this difference was not statistically significant (p = 0.184). Comparison of lipid parameters between the two groups showed that low-density lipoprotein cholesterol (LDL-C) levels were significantly higher in the dyslipidemia group (169.53 ± 8.95 mg/dL) compared with the non-dyslipidemia group (124.78 ± 3.39 mg/dL) (p < 0.001). High-density lipoprotein cholesterol (HDL-C) levels were significantly lower in the dyslipidemia group (35.97 ± 3.35 mg/dL) than in the non-dyslipidemia group (52.42 ± 5.23 mg/dL) (p < 0.001). Triglyceride (TG) levels were also significantly higher in the dyslipidemia group (166.88 ± 9.83 mg/dL) compared with the non-dyslipidemia group (137.84 ± 6.48 mg/dL) (p < 0.001). Similarly, total cholesterol (TC) levels were significantly higher in the dyslipidemia group (247.21 ± 18.92 mg/dL) compared with the non-dyslipidemia group (181.52 ± 12.22 mg/dL) (p < 0.001).

**Table 2:** The prevalence of clinical parameters with respect to history of dyslipidemia. Statistical analysis: Independent t-test and Mann–Whitney U test.

Variable	History of dyslipidemia	
	Yes, Mean±SD	No, Mean±SD
Age (years)	60.0±15.85	53.51±14.40
Weight (kg)	72.05±13.07	66.90±16.01
Height (inch)	68.09±10.88	64.67±7.43
Respiratory rate (cycles/min)	20.27±5.97	18.30±5.58
Heart rate beats/min	87.68±10.46	82.62±12.02
Random blood sugar (RBS) (mg/dL)	335.88±97.64	320.70±111.99
Lipid parameters		
Low-density lipoprotein (mg/dL)	169.53±8.95	124.78±3.39
High-density lipoprotein (mg/dL)	35.97±3.35	52.42±5.23
Triglycerides (mg/dL)	166.88±9.83	137.84±6.48
Total cholesterol (mg/dL)	247.21±18.92	181.52±12.22

Urinary symptoms

Table 3 shows the prevalence of urinary symptoms among patients with T2DM according to their history of dyslipidemia. Frequent urination was reported by 99 (58.2%) patients with dyslipidemia compared with 46 (27.1%) patients without dyslipidemia, showing a significant association (p < 0.001). Among patients who reported frequent urination, 60 (35.3%) in the dyslipidemia group and 78 (45.9%) in the non-dyslipidemia group experienced more than double the normal daytime frequency. Nocturia was slightly more common in patients with dyslipidemia (78 (45.9%)) than in those without dyslipidemia (61 (35.9%)); however, this difference was not statistically significant (p = 0.108). Regarding urine color, light-colored urine was observed in 79 (46.5%) patients with dyslipidemia compared with 129 (75.9%) patients without dyslipidemia. Dark yellow urine was reported by 76 (44.7%) patients with dyslipidemia versus 41 (24.1%) patients without dyslipidemia. Very dark or bloody urine was present in 15 (8.8%) patients with dyslipidemia, whereas none (0.0%) of the patients without dyslipidemia reported this symptom, indicating a statistically significant association (p < 0.001).

**Table 3:** The prevalence of urinary symptoms between T2DM patients with and without a history of dyslipidemia. T2DM: type 2 diabetes mellitus.

Variable	Category	History of dyslipidemia		Pearson chi square	degrees of freedom (df)	Effect size (Cramer’s V)	P-value
		Yes, n(%)	No, n(%)				
frequent urination	Yes	99(58.2%)	46(27.1%)	33.778	1	0.315	<0.001
	No	71(41.8%)	124(72.9%)				
If yes, then	Double the normal frequency during the day	60(35.3%)	78(45.9%)	19.476	2	0.239	0.108
	Nocturia	78(45.9%)	61(35.9%)				
	Less than normal frequency	32(18.8%)	31(18.2%)				
Urine color	Light-colored urine	79(46.5%)	129(75.9%)	37.489	2	0.332	<0.001
	Dark yellow urine	76(44.7%)	41(24.1%)				
	Very dark or bloody urine	15(8.8%)	0(0.0%)				

Visual and respiratory symptoms

Visual symptoms were more common among patients with dyslipidemia. Visual impairment was reported by 104 (61.2%) patients with dyslipidemia compared with 72 (42.4%) patients without dyslipidemia (p = 0.001). Blurred vision was also more frequent in the dyslipidemia group, reported by 78 (45.9%) patients compared with 47 (27.6%) patients without dyslipidemia (p < 0.001). Vision loss showed a similar pattern, affecting 75 (44.1%) patients with dyslipidemia and 63 (37.1%) patients without dyslipidemia (p = 0.003). Respiratory symptoms also varied between groups. Among patients reporting breathing difficulty, moderate symptoms were more prevalent in the dyslipidemia group (88 (51.8%)), whereas mild symptoms were more common in the non-dyslipidemia group (101 (59.4%)) (p < 0.001). Chest tightness or pressure was reported by 136 (80.0%) patients with dyslipidemia compared with 116 (68.2%) patients without dyslipidemia (p = 0.013). However, the severity of chest pain did not differ significantly between the groups (p = 0.264), as shown in Table 4.

**Table 4:** Comparison of visual disturbances and respiratory symptoms between T2DM patients with and without a history of dyslipidemia. T2DM: type 2 diabetes mellitus.

Variable	Category	History of dyslipidemia		Pearson chi square	degrees of freedom (df)	Effect size (Cramer’s V)	P-value
		Yes, n(%)	No, n(%)				
Visual impairment	Yes	104(61.2%)	72(42.4%)	12.062	1	0.188	0.001
	No	66(38.8%)	98(57.6%)				
Blurred vision	Yes	78(45.9%)	47(27.6%)	12.158	1	0.189	<0.001
	No	92(54.1%)	123(72.4%)				
Vision loss	Yes	75(44.1%)	63(37.1%)	8.663	1	0.16	0.003
	No	95(55.9%)	107(62.9%)				
Difficulty breathing if yes	Mild	52(30.6%)	101(59.4%)	28.542	2	0.29	<0.001
	Moderate	88(51.8%)	51(30.0%)				
	Severe	30(17.6%)	18(10.6%)				
Do you ever feel chest tightness or pressure	Yes	136(80.0%)	116(68.2%)	6.133	1	0.134	0.013
	No	34(20.0%)	54(31.8%)				
Severity of chest pain	Improves with rest	89(52.4%)	99(58.2%)	2.665	2	0.089	0.264
	Need pain-relieving medication	67(39.4%)	53(31.2%)				
	Requires a hospital visit	14(8.2%)	18(10.6%)				

Other symptoms

As shown in Table 5, tingling or numbness in the hands or feet was reported by 103 (60.6%) patients with dyslipidemia and 94 (55.3%) patients without dyslipidemia, showing no significant association (p = 0.323). Burning pain in the legs or feet was more common in the dyslipidemia group, reported by 116 (68.2%) patients compared with 94 (55.3%) patients without dyslipidemia (p = 0.014). Muscular pain or cramps were also significantly more frequent among patients with dyslipidemia, affecting 160 (94.1%) patients compared with 142 (83.5%) patients without dyslipidemia (p = 0.002). Loss of appetite and insomnia showed no statistically significant differences between the groups (p = 0.318 and p = 0.384, respectively). Increased thirst was markedly more common in patients with dyslipidemia, reported by 100 (58.8%) patients compared with 64 (37.6%) patients without dyslipidemia (p < 0.001). Fatigue was significantly more prevalent in the dyslipidemia group, affecting 160 (94.1%) patients, compared with 141 (82.9%) patients in the non-dyslipidemia group (p = 0.001). Increased hunger did not differ significantly between the groups (p = 0.322). Feeling tired or weak was significantly more common among patients with dyslipidemia, reported by 148 (87.1%) patients compared with 118 (69.4%) patients without dyslipidemia (p < 0.001). Irritability or mood changes were also significantly higher in the dyslipidemia group, reported by 145 (85.3%) patients compared with 123 (72.4%) patients without dyslipidemia (p = 0.003).

**Table 5:** The prevalence of others symptoms between T2DM patients with and without a history of dyslipidemia. T2DM: type 2 diabetes mellitus.

Variable	Category	History of dyslipidemia		Pearson chi square	degrees of freedom df	Effect size (Cramer’s V)	P-value
		Yes, n(%)	No, n(%)				
Tingling or numbness in the hands or feet	Yes	103(60.6%)	94(55.3%)	0.978	1	0.054	0.323
	No	67(39.4%)	76(44.7%)				
Burning pain in your legs or feet	Yes	116(68.2%)	94(55.3%)	6.028	1	0.133	0.014
	No	54(31.8%)	76(44.7%)				
Muscular pain or cramps in your legs or feet	Yes	160(94.1%)	142(83.5%)	9.599	1	0.168	0.002
	No	10(5.9%)	28(16.5%)				
Loss of appetite	Yes	107(62.9%)	98(57.6%)	0.995	1	0.054	0.318
	No	63(37.1%)	72(42.4%)				
Insomnia	Yes	83(48.8%)	75(44.1%)	0.757	1	0.047	0.384
	No	87(51.2%)	95(55.9%)				
Increased thirst	Yes	100(58.8%)	64(37.6%)	15.266	1	0.212	<0.001
	No	70(41.2%)	106(62.4%)				
Fatigue	Yes	160(94.1%)	141(82.9%)	10.456	1	0.175	0.001
	No	10(5.9%)	29(17.1%)				
Increased hunger	Yes	48(28.2%)	40(23.5%)	0.981	1	0.054	0.322
	No	122(71.8%)	130(76.5%)				
Feel tired and weak occasionally	Yes	148(87.1%)	118(69.4%)	15.546	1	0.214	<0.001
	No	22(12.9%)	52(30.6%)				
Feel irritability or having other mood changes	Yes	145(85.3%)	123(72.4%)	8.528	1	0.158	0.003
	No	25(14.7%)	47(27.6%)				

## Discussion

Our study revealed a striking gender disparity in dyslipidemia distribution, with male predominance in the dyslipidemia group (117 (68.8%)) compared with the non-dyslipidemia group (43 (25.3%)) (p < 0.001). This finding presents a notable contrast to existing literature, where a 2024 study reported a more balanced gender distribution, with males comprising 42.08% of patients with dyslipidemia [13]. Even more striking is the comparison with a 2022 hospital-based study of hypertensive patients, which demonstrated a higher prevalence of dyslipidemia among women (72%) compared with men (54%) [14]. These disparate findings could be attributed to several factors, including potential selection bias in our study population, demographic variations, or unique characteristics of our health care setting. The extreme gender disparity observed in our findings may reflect specific recruitment patterns, health care-seeking behaviors, or referral patterns within our study context. Additionally, our results diverge from a 2023 study on familial hypercholesterolemia, which noted a higher prevalence of obesity among women, potentially influencing lipid profiles differently from those in our population [15]. These contrasting findings highlight the need for more comprehensive gender-specific research in dyslipidemia, particularly with respect to screening protocols, risk assessment, and treatment strategies.

Our study findings regarding smoking, physical activity, and diabetes duration demonstrate significant associations with dyslipidemia that both align with and extend current literature. The observed smoking prevalence in the dyslipidemia group (45.3% vs 17.1%, p < 0.001) mirrors findings reported in a 2024 study that identified comparable rates, reinforcing the robust association between smoking and dyslipidemia [16]. The physical activity findings in our study (61.2% vs 73.5%, p = 0.015) align closely with another 2024 study, which similarly demonstrated lower physical activity levels among patients with dyslipidemia [17]. This consistency across studies strengthens the evidence for a sedentary lifestyle as a significant contributing factor to lipid abnormalities. However, Katundu et al. (2022) expanded on these findings by highlighting the role of genetic predispositions and dietary habits, suggesting that while the identified risk factors are significant, they represent only part of a more complex pathophysiological picture [18].

The cardiovascular risk profile of patients with dyslipidemia in our study shows notable patterns that both align with and extend current research findings. Our data indicate significant differences in vital signs between patients with and without dyslipidemia, particularly respiratory and heart rates, suggesting increased cardiovascular strain. This observation aligns with a 2023 case-control study on coronary heart disease risk, which demonstrated that dyslipidemia, particularly when combined with hypertension, significantly increases cardiovascular risk. While that study focused primarily on the direct relationship between dyslipidemia and coronary heart disease, our findings add relevant physiological parameters to this risk profile, particularly the observation of elevated heart rates (87.68 beats/min) and respiratory rates (20.27 cycles/min) among patients with dyslipidemia [19].

Our study revealed significant associations between dyslipidemia and various visual disturbances, with findings that both corroborate and extend current research. The higher prevalence of visual impairment observed in the dyslipidemia group (61.2% vs 42.4%, p = 0.001) aligns closely with findings reported by Hamedani et al. in 2024, suggesting a consistent pattern of visual involvement in this population [20].

The relationship between dyslipidemia and visual health appears to be multifaceted, as demonstrated by recent research. While our study focused on functional visual disturbances, a 2018 study provided important context by establishing links between dyslipidemia and dry eye disease (DED), particularly in women [21]. This association was further elaborated by Serrano-Morales et al. in 2024, who described mechanisms through which elevated cholesterol and LDL levels impair tear film stability and meibomian gland function. These findings suggest that the visual disturbances observed in our study may be partially attributable to underlying surface eye conditions [22].

Our findings take on additional significance when considered alongside a 2024 study by Habbak et al. examining the relationship between dyslipidemia and glaucoma. The high prevalence of vision loss observed in the dyslipidemia group (44.1%) may reflect not only functional impairment but also potentially irreversible structural changes [23]. This suggests that visual disturbances in patients with dyslipidemia should be viewed not merely as symptoms but as possible indicators of progressive ocular damage.

Our study revealed unexpected patterns in pain perception among patients with dyslipidemia that both challenge and extend current understanding of lipid-pain relationships. The higher prevalence of burning pain in the legs or feet among patients with dyslipidemia (68.2% vs 55.3%, p = 0.014), along with increased muscular pain and cramps (94.1% vs 83.5%, p = 0.002), presents an interesting contrast to traditional assumptions regarding dyslipidemia and pain sensitivity. These findings partially align with research by Wang et al. (2021), which demonstrated an association between higher HDL levels and reduced chronic nonmalignant pain in elderly patients, suggesting a potential protective mechanism through lipid metabolism [24].

However, our results present an intriguing paradox when considered alongside findings by Nk et al. (2013), which indicated that low HDL and high LDL levels correlate with increased pain severity. The higher prevalence of pain symptoms observed in our dyslipidemia population, despite presumably adverse lipid profiles, suggests that the relationship between lipid metabolism and pain perception may be more complex than previously recognized. This discrepancy may be explained by differences in pain mechanisms, variations in study populations, or the presence of confounding variables not previously considered [25].

This study has certain limitations, including a moderate sample size and recruitment from a limited demographic, which may restrict the generalizability of the findings. Potential confounding factors such as dietary habits, medication use, and the duration or level of control of diabetes and dyslipidemia were not fully controlled, which could have influenced the observed associations. Future studies involving larger, more diverse populations and adjustment for these variables are recommended to validate and expand upon these results.

## Conclusions

This study concluded that patients with type 2 diabetes mellitus and dyslipidemia had a higher prevalence of visual disturbances, urinary symptoms, fatigue, musculoskeletal pain, and other systemic complaints compared with non-dyslipidemic patients. Significant differences were observed in blurred vision, chest discomfort, increased thirst, fatigue, and mood changes. These findings indicate that dyslipidemia is associated with more pronounced clinical features in T2DM, underscoring the need for careful monitoring and management of these patients.
